# Impact of Body Mass Index Variability on Kidney Disease Progression in a Large Type 1 Diabetes Cohort

**DOI:** 10.1002/dmrr.70148

**Published:** 2026-03-19

**Authors:** Murat Ozdede, Panagiotis Pavlou, Marta Avataneo, Stephen Thomas, Salma Ayis, Janaka Karalliedde

**Affiliations:** ^1^ School of Cardiovascular and Metabolic Medicine and Sciences King's College London London UK

**Keywords:** BMI variability, diabetic kidney disease, Kidney disease progression, metabolic cycling, type 1 diabetes

## Abstract

**Aims:**

Body mass index (BMI) variability an index of ‘metabolic cycling’ predicts kidney disease in type 2 diabetes, but studies in people with type 1 diabetes (pwT1DM) are scarce. We examined the relationship between BMI variability and diabetic kidney disease (DKD) progression in an ethnically diverse cohort of pwT1DM.

**Methods:**

We analysed 3270 pwT1DM (52% female, 80% white) with baseline eGFR ≥ 45 mL/min/1.73 m^2^ and ≥ 6 BMI measurements, attending two university hospital clinics (between 2004 and 2018). BMI variability was assessed using standard deviation (SD), visit‐adjusted SD, variability independent of the mean (VIM), and average real variability (ARV). The primary endpoint was ≥ 50% eGFR decline with final eGFR < 30 mL/min/1.73 m^2^. Multivariable and competing risk analyses (with mortality as competing risk) were performed.

**Results:**

Over a median of 9.6 years, 179 (5.5%) people reached the primary endpoint. Those who reached the primary endpoint had higher BMI variability, with baseline higher age, HbA1c, systolic blood pressure, albuminuria levels, and were more often of African‐Carribean ethnicity as compared to those who did not. In multivariable models and competing risk analyses, all BMI variability indices were independent risk‐factors for the primary endpoint. Visit‐adjusted SD (HR 2.43, 95% CI 1.93–3.05) and ARV (HR 1.7, 95% CI 1.44–2.01) were the strongest BMI variability indices associated with the primary endpoint.

**Conclusion:**

BMI variability is an independent predictor of DKD progression in pwT1DM. Further studies are required to elucidate the underlying mechanisms of our observations and explore if addressing BMI variability can translate to clinical benefits in DKD.

## 1 Introduction

Diabetic kidney disease (DKD) is a well‐defined complication of diabetes mellitus affecting up to 40% of people with diabetes and constitutes a spectrum ranging from early stages such as hyperfiltration, to end‐stage kidney disease [1]. In people with type 1 diabetes (pwT1D), metabolic and haemodynamic changes drive disease onset and progression [1, 2]. Traditional modifiable risk factors for DKD progression in pwT1DM include HbA1c, blood pressure, albuminuria and features of ‘metabolic syndrome’ [3, 4].

Most of these factors are not static in nature and can vary significantly over time due to many reasons. There is evidence that HbA1c variability strikingly impacts on the onset and progression of microvascular complications including DKD [5]. Furthermore there is also a growing recognition and appreciation in the literature that the magnitude of fluctuations/variability in certain parameters is as crucial as their cross‐sectional or mean values in predicting future risk of diabetes complications [6].

There is a biological plausibility that increased BMI and body fat contribute to DKD pathogenesis by promoting hyperfiltration via haemodynamic changes and impairing microvascular function through altering metabolic pathways [4, 7, 8, 9]. Thus, weight control to an appropriate extent might be protective, justifying weight loss in people who are overweight or obese as a treatment principle for renal protection, and this is supported by recent evidence that suggests weight loss via pharmacological or non‐pharmacological methods can improve DKD markers and risk [10]. However, weight loss attempts can often be followed by weight regain within years leading to repeated cycles of loss and regain [11]. This concept of ‘metabolic cycling’, as indicated by BMI variability, is associated with adverse outcomes such as mortality, cardiovascular disease and microvascular complications including DKD in people with type 2 diabetes (T2DM) [11, 12, 13, 14, 15, 16, 17, 18, 19]. A recent retrospective cohort study using weight data collected from the Diabetes Control and Complications Trial (DCCT)/Epidemiology of Diabetes Interventions and Complications (EDIC) [DCCT‐EDIC] cohort, demonstrated that weight variability was an independent risk factor for kidney disease progression defined primarily as > 40% drop in estimated glomerular filtrate rate (eGFR) [20]. This cohort, which included data in children and pregnancy, was not ethnically diverse and the number of events of advanced kidney disease (eGFR < 30 mL/min/1.73 m^2^) was modest.

The aim of our study, conducted in a contemporary, ethnically diverse cohort of urban‐dwelling adults with type 1 diabetes, was to examine the relationship between BMI variability and the risk of clinically significant kidney disease defined as > 50% drop in eGFR with a final eGFR < 30 mL/min/1.73 m^2^.

### 1.1 Research Design and Methods

Full details of the study cohort and related detailed methods have been described in detail in previously published work [21]. In brief, anonymised electronic health records/data from people attending routine outpatient care between 2004 and 2018 at two large university hospitals in south London were collected. Information such as date of birth, date of death (when applicable), sex, ethnicity (self‐reported), systolic/diastolic blood pressure measurements (SBP/DBP), laboratory measurements such as serum creatinine, urine albumin‐creatinine ratio, HbA1c, and height and weight, taken at each clinic visit using calibrated equipment and standardised methods, were extracted from electronic health records. The primary endpoint of our study was progression of kidney disease defined as a ≥ 50% decline in eGFR from baseline and a final eGFR < 30 mL/min/1.73 m^2^. Inclusion criteria required a baseline eGFR of ≥ 45 mL/min/1.73 m^2^ and the availability of at least six BMI records during the study period. Exclusion criteria were pregnancy, absent follow‐up eGFR, < 6 BMI measurements or known non‐diabetic kidney disease. Serum creatinine, eGFR and other biochemical or clinical measures from acute admissions were excluded. Death before DKD progression was recorded and treated as a competing risk in survival models but censored in descriptive analyses.

Serum creatinine measurements were used to calculate eGFR values using the updated Chronic Kidney Disease Epidemiology Collaboration (CKD‐EPI) equation [22]. All laboratory tests were performed by the same central provider. The date of first serum‐creatinine is the inclusion‐date, with baseline variables defined within a 2‐year timespan. The final follow‐up date was the date of the kidney‐disease progression (if applicable), date of death, or last eGFR measurement, whichever occured the earliest. Socioeconomic status was measured through the validated Index of Multiple Deprivation (IMD) deciles, which are based on the United Kingdom Office of National Statistics data on socio‐economic indices, metrics and the individual post‐code. The IMD is stratified into population deciles, whereby 1 indicates the highest deprivation and 10 is most affluent [23].

#### 1.1.1 Statistical Analysis

BMI variability was assessed by the following methods: [1] standard deviation (SD), [2] visit‐adjusted SD (adj‐SD) via $\text{SD}÷\surd \frac{\text{visit}\;\text{count}}{\left(\text{visit}\;\text{count}-1\right)}$, [3] Variability Independent of the Mean (VIM) via ${\text{adj}-\text{SD}\times \left(\bar{\frac{X}{X_{0}}}\right)}^{-\beta }$, where *β* is set to a scaling parameter value of 2, and [4] Average Real Variability via $\frac{1}{N-1}\sum_{i=1}^{N-1}\left|x_{i+1}-x_{i}\right|$. VIM was chosen for its strength against baseline biases, while ARV inherently adjusts for differences in visit counts. Additionally, BMI variability quartiles (Q1: stable, Q2: mild fluctuators, Q3: moderate fluctuators, Q4: severe fluctuators) were used to stratify patients.

Log‐transformation was applied to variability indices and ACR data due to skewed distributions. Descriptive statistics are reported as mean ± standard deviation for normally distributed or log‐transformed continuous variables, median (interquartile range) for non‐normal continuous variables and frequency (*n*, %) for categorical variables. Group comparisons were performed using independent *t*‐tests for normally distributed variables, Mann‐Whitney U tests for non‐parametric variables, and *χ* ^2^ tests for categorical variables if they met the *χ* ^2^ assumptions; otherwise, Fisher's exact test was applied. Variables with < 30% missing data within the baseline dataset were included in the analysis. Missing baseline data for continuous variables were imputed using predictive mean matching via the Multiple Imputation by Chained Equations (MICE) [24] package in R; however, certain critical parameters defining the study, such as BMI and eGFR, were not imputed.

Time‐to‐event analyses utilised univariate cause‐specific hazard models to assess DKD progression, incorporating traditional risk factors identified in previous studies alongside BMI variability indices [21]. Multivariate cox proportional hazard models included baseline covariates including, self‐reported ethnicity stratified into African‐Caribbean and non‐African Caribbean heritage and baseline eGFR, HbA1c, log ACR, age and SBP, with BMI variability indices added individually to each model. Comparisons of model performance were conducted using concordance indices, and Akaike Information Criterion (AIC) values (details in Supporting Information S2: Tables). Socioeconomic status (Index of Multiple Deprivation [IMD] decile) was additionally included in sensitivity models to assess potential confounding (Supporting Information S2: Tables). Endpoint incidences were visualised using cumulative incidence plots (Supporting Information S1: Figure SF1). Time‐dependent Area Under the Curve (AUC) analyses were performed to evaluate and visualise model performance over time (Supporting Information S1: Figure SF2). Baseline model and models with additional two top ranking variability indices were performed via Fine‐Gray competing risk models to account for death as a competing risk for DKD progression [25]. Additionally, BMI variability quartiles were compared through Kaplan‐Meier failure probability plots and log‐rank tests were employed to compare quartiles.

All statistical analyses and cumulative incidence plots were performed using RStudio (version 2024.04.2.0). Time‐dependent ROC curves and Kaplan‐Meier plots were generated using Python (Spyder IDE) to enhance visualisation. Statistical significance was determined by two‐sided *p* values < 0.05. This retrospective study was conducted in line with local audit protocols using existing anonymised routine clinical data accessed directly by the clinical team approved by the hospital data governance committees.

## 2 Results

The cohort anlaysed in this study included a total number of 3270 pwT1DM with ≥ 6 BMI measurements from baseline. Of the 3270 people, 1710 (52.3%) were female, and their mean age (± standard deviation) was 37.3 (± 14.1) years. Mean baseline BMI was 25.2 ± 4.6 and eGFR of 91.5 ± 23.8, with an overall median (IQR) follow‐up period of 9.6 (6.45) years. Of the cohort ∼80% were of White origin with 12.2% of Black African‐Caribbean heritage, 2.9% Asian and 5.1% Other ethnicities (which included mixed race people). Overall, 179 (5.5%) of people progressed to the primary endpoint and 88 (2.7%) people died within the study period of 14 years.

At baseline, people who progressed to DKD were significantly older and exhibited higher levels of HbA1c, systolic blood pressure, triglycerides, and albumin‐to‐creatinine ratio (ACR) compared with those who did not progress (Table 1). While baseline BMI was comparable between the groups, log‐transformed BMI variability indices were significantly elevated in those who experienced the primary outcome (Table 1). BMI log‐SD and VIM was markedly higher in those with DKD progression (0.98 ± 0.33 vs. 0.76 ± 0.32, *p* < 0.001). To account for differences in visit counts across the groups, visit count‐adjusted parameters were evaluated. Consistent with other above indices, log‐adj‐SD and ARV BMI variability also showed a similar, statistically significant differences (Table 1).

**TABLE 1:** Baseline characteristics of 3270 people with type 1 diabetes and those with and without progression of kidney disease.

Characteristics	Total cohort *n* = 3270	No progression *n* = 3091 (94.5%)	Progression *n* = 179 (5.5%)	*p* values
Sex^a^				0.8
Female	1710 (52.3%)	1618 (94.6%)	92 (5.4%)	
Male	1560 (47.7%)	1473 (94.4%)	87 (5.6%)	
Age	37.3 (± 14.1)	37 (± 13.8)	43.5 (± 16.8)	< 0.001
Ethnicity^a^				< 0.001
Caucasian	2611 (79.8%)	2489 (95.3%)	124 (4.7%)	
African Caribbean	398 (12.2%)	352 (88.4%)	46 (11.6%)	
Asian	95 (2.9%)	92 (96.8%)	3 (3.2%)	
Mixed heritage or others	166 (5.1%)	160 (96.4%)	6 (3.6%)	
Duration of diabetes (years)	12.7 (± 13)	12.6 (± 12.8)	14 (± 14.6)	0.22
Retinopathy^a^				0.09
No	2101 (64.3%)	1997 (95%)	104 (5%)	
Yes	1169 (35.7%)	1094 (93.5%)	75 (6.5%)	
BMI (kg/m^2^)	25.2 (± 4.6)	25.2 (± 4.6)	25.8 (± 5.4)	0.14
eGFR (ml/min/1.73 m^2^)	91.5 (± 23.8)	92.2 (± 23.6)	78.5 (± 22.1)	< 0.001
Weight (kg)	73 (± 15.9)	73.2 (± 15.8)	72.8 (± 17.02)	0.5
Systolic blood pressure (mmHg)	123.5 (± 15.7)	123.3 (± 15.5)	128 (± 17.5)	< 0.001
Diastolic blood pressure (mmHg)	73.6 (± 9.21)	73.5 (± 9.1)	74.2 (± 10.4)	0.33
HbA1C (mmol/mol)	73.5 (± 24.3)	72.8 (± 23.7)	85.7 (± 30.8)	< 0.001
Cholesterol (mmol/L)	4.67 (± 1)	4.67 (± 1.6)	4.7 (± 1.3)	0.72
HDL (mmol/L)	1.63 (± 0.48)	1.63 (0.47)	1.61 (± 0.6)	0.66
LDL (mmol/L)	2.5 (± 0.84)	2.52 (± 0.83)	2.41 (1.02)	0.15
Triglycerides (mmol/L)	1.24 (± 0.9)	1.23 (± 0.86)	1.57 (± 1.27)	< 0.001
Albuminuria Classes^a^				< 0.001
A1	462 (14.1%)	445 (14.4%)	17 (9.5%)	
A2	1536 (47%)	1477 (47.8%)	59 (33%)	
A3	1272 (38.9%)	1169 (37.8%)	103 (57.5%)	
IMD_DECILE (IQR)^b^	3 (3)	3 (3)	3 (3)	0.9
Follow up time (IQR)^b^ (years)	9.6 (6.45)	9.8 (6.4)	6.9 (6.3)	< 0.001
Variability indices				
log‐SD	0.77 (0.32)	0.76 (± 0.32)	0.98 (± 0.33)	< 0.001
log_adj‐SD	1.61 (0.58)	1.6 (± 0.57)	2 (± 0.55)	< 0.001
log‐VIM	1.65 (0.69)	1.63 (± 0.67)	2.1 (± 0.73)	< 0.001
log‐ARV	0.55 (0.22)	0.54 (± 0.22)	0.68 (± 0.2)	< 0.001

Abbreviations: A1, albuminuria below 2.99 mg/mmol; A2, albuminuria 3–29.99 mg/mmol; A3, albuminuria > 30 mg/mmol; BMI, Body Mass Index; DKD, Diabetic Kidney Disease; HbA1c, Glycosylated haemoglobin; HDL, High‐density lipoprotein; IMD, Index of Multiple Deprivation; LDL, Low‐density lipoprotein; log_adj‐SD, Log‐transformed visit‐adjusted standard deviation of BMI; log_ARV, Log‐transformed average real variability for BMI; log_SD, Log‐transformed standard deviation of BMI; log_VIM, Log‐transformed variability independent of the mean for BMI.

^a^ Parameters are presented as percentages and counts and comparison test is chi‐square or Fisher's exact test.

^b^ Parameters are presented as medians with interquartile ranges (IQR), comparison test is Mann Whitney *U* test.

When comparing baseline characteristics across ethnicities, stratified as African‐Caribbean and non‐African‐Caribbean, results were consistent with earlier studies, and baseline BMI was comparable between the groups (25.3 ± 5.2 vs. 25.2 ± 4.5, *p* = 0.8) [26, 27] (Table 1). However, BMI variability indices estimated through all four methods were significantly higher in African‐Caribbean participants compared to non‐African‐Caribbean participants (Supporting Information S2: Table S1). Specifically, log‐SD‐BMI was 0.84 ± 0.32 versus 0.76 ± 0.32 (*p* < 0.001), log‐adj‐SD‐BMI was 1.75 ± 0.6 versus 1.6 ± 0.57 (*p* < 0.001), log‐VIM‐BMI was 1.8 ± 0.68 versus 1.64 ± 0.68 (*p* < 0.001), and log‐ARV‐BMI was 0.57 ± 0.2 versus 0.54 ± 0.2 (*p* < 0.001).

Correlation tests between BMI variability indices revealed strong and significant intercorrelations among all four indices, as expected (Supporting Information S2: Table S2). All indices also showed weak but significant positive correlations with baseline HbA_1c_, highlighting a potential link between BMI variability and glycaemic control (Supporting Information S2: Table S2).

Multivariable hazard models incorporating baseline covariates with BMI variability indices demonstrated significant improvements in model performance metrics. While baseline risk factors, including higher age, lower eGFR, African Caribbean ethnicity, higher HbA1c, and elevated ACR levels, were strongly associated with the primary endpoint (Table 2), among all models tested, the model incorporating log‐adj‐SD as a BMI variability measure exhibited the best Akaike Information Criterion (AIC) value (2549.2 vs. baseline value 2591.9; Supporting Information S2: Table S3), indicating superior overall performance. Deprivation, as measured by IMD deciles, did not differ between participants who did and did not progress (Table 1) and was not associated with DKD progression in cause‐specific Cox models (HR ∼1.01–1.03 per decile; *p* > 0.4). The inclusion of IMD also did not change the significant association between BMI variability and DKD progression (Supporting Information S2: Table S4). Time‐dependent AUC plots (Supporting Information S1: Figure SF2) revealed that models including ARV demonstrated consistently strong predictive performance over time, particularly during the later years of follow‐up, reflecting its stability and effectiveness in long‐term risk prediction and justifying the use of two separate indices, log‐adj‐SD and log‐ARV, for final competing risk models.

**TABLE 2:** Impact of BMI variability evaluated by four distinct methods and other known traditional risk factors, through multivariate Cox regression modelling, on kidney disease progression in people with type 1 diabetes.

	Cox model 1: Standard deviation		Cox model 2: Visit‐adjusted standard deviation		Cox model 3: Variability independent of mean		Cox model 4: Average real variability	
Variables	HR (95% CI)	*p*‐value	HR (95% CI)	*p*‐value	HR (95% CI)	*p*‐value	HR (95% CI)	*p*‐value
Variability indices								
log SD	3.91 (2.63–5.82)	< 0.001						
log adj‐SD			2.38 (1.85–3.06)	< 0.001				
log VIM					1.81 (1.49–2.20)	< 0.001		
log ARV							5.09 (3.08–8.40)	< 0.001
Covariates								
Baseline age	1.03 (1.01–1.04)	< 0.001	1.03 (1.01–1.04)	< 0.001	1.03 (1.01–1.04)	< 0.001	1.02 (1.01–1.04)	< 0.001
Baseline eGFR	0.98 (0.98–0.99)	< 0.001	0.98 (0.97–0.99)	< 0.001	0.98 (0.98–0.99)	< 0.001	0.99 (0.98–0.99)	< 0.001
Baseline HbA1c	1.02 (1.01–1.03)	< 0.001	1.02 (1.02–1.03)	< 0.001	1.02 (1.02–1.03)	< 0.001	1.02 (1.02–1.03)	< 0.001
log ACR	1.28 (1.11–1.46)	< 0.001	1.31 (1.15–1.50)	< 0.001	1.32 (1.15–1.50)	< 0.001	1.27 (1.11–1.46)	< 0.001
Baseline systolic blood pressure	1.01 (1.00–1.02)	0.068	1.01 (1.00–1.02)	0.072	1.01 (1.00–1.02)	0.191	1.01 (1.00–1.02)	0.076
African Caribbean ethnicity	1.86 (1.32–2.63)	< 0.001	1.80 (1.27–2.55)	< 0.001	1.79 (1.26–2.54)	< 0.001	1.88 (1.32–2.67)	< 0.001

Abbreviations: eGFR, estimated Glomeruler Filtration Rate; HbA1c, Glycosylated haemoglobin; HDL, High‐density lipoprotein; LDL, Low‐density lipoprotein; log‐ACR, Log‐transformed Albumin‐creatinine ratio; log adj‐SD (visit‐adjusted), Log‐transformed visit‐adjusted standard deviation of BMI; log_ARV, Log‐transformed average real variability for BMI; log_SD, Log‐transformed standard deviation of BMI; log_VIM, Log‐transformed variability independent of the mean for BMI.

In multivariable Fine–Gray competing risk models (Table 3), treating death as a competing event, higher BMI variability remained independently associated with the primary kidney endpoint. Each 1‐unit increase in log(visit‐adjusted SD of BMI) was associated with a higher subdistribution hazard (HR 2.43, 95% CI 1.93–3.05), after adjustment for age, HbA1c, systolic blood pressure, baseline eGFR, albuminuria, and ethnicity. Similar findings were observed for log ARV of BMI (HR 5.18, 95% CI 3.31–8.11).

**TABLE 3:** Impact of BMI variability and traditional risk factors on kidney disease progression and death as competing risks in people with type 1 diabetes.

	Baseline model		Model with visit‐adjusted SD		Model with ARV	
Variables	Hazard ratio (95% CI)	*p* value	Hazard ratio (95% CI)	*p* value	Hazard ratio (95% CI)	*p* value
Age	1.02 (1.01–1.03)	< 0.001	1.03 (1.01–1.04)	< 0.001	1.02 (1.01–1.03)	< 0.001
eGFR	0.99 (0.98–1)	0.002	0.98 (0.97–0.99)	< 0.001	0.99 (0.98–0.99)	0.002
HbA1c	1.02 (1.02–1.03)	< 0.001	1.21 (1.01–1.03)	< 0.001	1.22 (1.02–1.03)	< 0.001
Systolic pressure	1.01 (1.00–1.02)	0.11	1.01 (1.00–1.02)	0.08	1.02 (1.02–1.03)	0.04
African Caribbean ethnicity	1.77 (1.25–2.52)	0.001	1.81 (1.29–2.52)	< 0.001	1.74 (1.25–2.43)	0.002
log‐ACR	1.33 (1.16–1.52)	< 0.001	1.31 (1.14–1.5)	< 0.001	1.28 (1.11–1.48)	< 0.001
log‐adj‐SD BMI			2.43 (1.93–3.05)	< 0.001		
log‐ARV BMI					5.18 (3.31–8.11)	< 0.001

Abbreviations: eGFR, estimated Glomerular Filtration Rate; log‐ACR, Log‐transformed Albumin‐creatinine ratio; log_ARV, Log‐transformed average real variability for BMI; log_SD (visit‐adjusted), Log‐transformed visit‐adjusted standard deviation of BMI.

Revisiting findings from our earlier study [21], the increased risk of the primary outcome in individuals of African‐Caribbean ethnicity was reaffirmed as independent of other traditional risk factors and BMI variability (Table 3).

**FIGURE 1:**
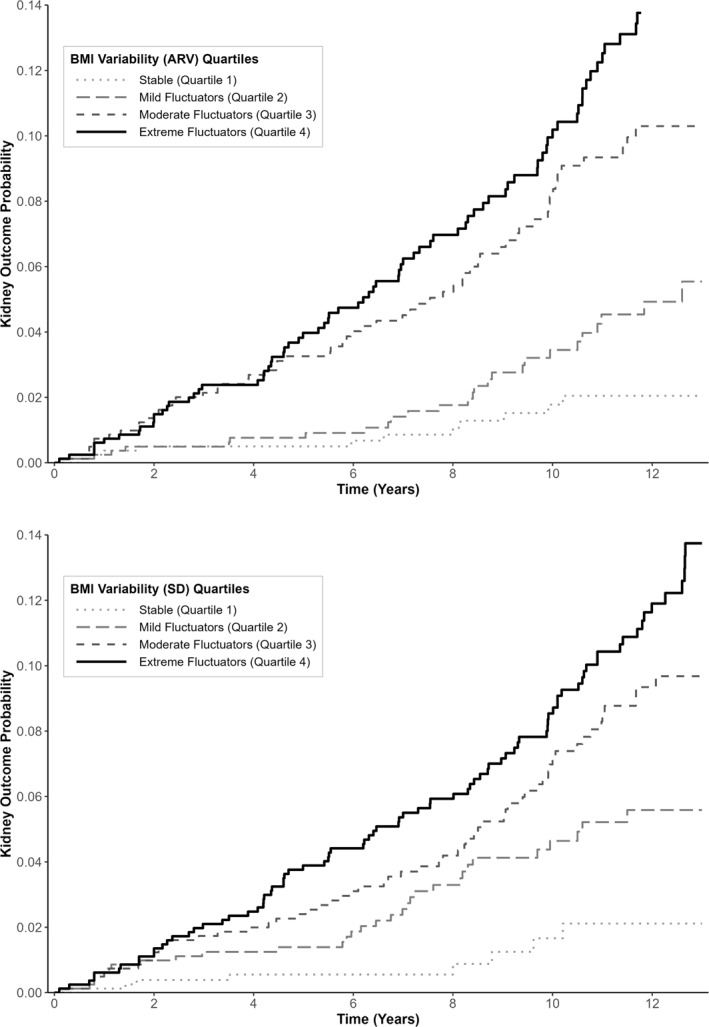
Kaplan‐Meier failure probability plots stratified by BMI variability quartiles, based on visit‐adjusted SD and ARV parameters, demonstrated clear differences in the kidney disease progression outcome.

Kaplan‐Meier plots (Figure 1) stratified by BMI variability quartiles, based on visit‐adjusted SD and ARV parameters, demonstrated clear differences in primary kidney outcome (Figure 1). People classified as having the highest BMI variability ‘Extreme Fluctuators’ (Quartile 4) had the highest risk of kidney disease progression. In contrast, people with less BMI variability ‘Stable’ category (Quartile 1) exhibited the most favourable outcomes, with minimal decline in kidney function. More specifically, people with the greatest BMI variability, ‘Extreme Fluctuators’ (Q4), had a significantly greater decline in kidney outcome probability over time compared to the ‘Stable’ category (log‐rank test, *χ* ^2^ = 38.7, *p* < 0.001). At 12 years, the cumulative incidence of the primary endpoint was 11.9% in Q4 compared to only 2.1% in Q1 (Stable group) (Supporting Information S1: Figure SF1), further illustrating the substantial impact of BMI variability on kidney disease progression in pwT1DM.

## 3 Conclusions

We observed that in a large, ethnically diverse cohort of adult pwT1DM, BMI variability was associated with DKD progression and this relationship remained significant even after adjusting for traditional risk factors such as age, baseline eGFR, baseline BMI, HbA1c, blood pressure, and ethnicity. To our knowledge, this study is unique as it focused on real world data from a cohort that is contemporaneous and ethnically diverse. Moreover, the kidney disease endpoint we utilised (> 50% fall in eGFR with final eGFR < 30 mL/min/1.73 m^2^) was indicative of severe DKD progression and of note, BMI variability remained significant in models with death as competing factor.

BMI and weight variability have been linked to macro‐ and microvascular events, cardiac arrhythmia and all‐cause mortality [14, 15, 16, 17, 18, 28, 29]; however, most of these studies have been in people without diabetes. A recent retrospective observational study in pwT1DM from the DCCT/EDIC historical cohort demonstrated the impact of weight variability (measured using three distinct indices) on a range of kidney outcomes, primarily a > 40% fall in eGFR from baseline [20]. These results on the role of weight variability (primarily assessed by VIM of weight) in pwT1DM on kidney endpoints, support our observations in a more contemporaneous real‐world setting. There are several notable differences between our study and this study based on the DCCT/EDIC cohort with regard to study population and methods. The study based on the DCCT/EDIC cohort included children and pregnant people, defined the renal endpoint as fall in eGFR > 40% from baseline, measured the role of weight variability rather than BMI and did not perform competing risk analyses of mortality, which can be an important confounder. Our study included only adult people, excluded pregnancy, explored the role of BMI variabilty rather than weight and our primary endpoint was defined as > 50% fall in eGFR with final eGFR < 30 mL/min/1.73 m^2^. We further examined the role of BMI variability on our primary endpoint with mortality as a competing risk in our analyses. Another notable difference is that the DCCT/EDIC cohort included data on treatment as a covariate, which we were unable to do as we lacked this data as well as related information on medication adherence in our electronic health records. There is evidence from the DCCT/EDIC data and other studies that intensive insulin therapy has effects on weight gain and that hypoglycaemia also influences weight cycling. Indeed the recent DCCT/EDIC study described above that investigated the effect of weight variability on kidney outcomes, excluded weight data in the first year of the DCCT trial for these reasons [20]. Data on reno‐protective therapies such as ACE‐inhibitor use were included in sensitivity analyses for the EDIC phase of the study where available and did not appear to influence the role of weight variability on kidney endpoints [20].

Apart from this recent study, data on the role of weight or BMI variability on kidney outcomes are sparse for people living with diabetes. One of the largest studies to date in a community based cohort of people living in South Korea demonstrated that weight variability was associated with rapid kidney function decline (defined as eGFR fall > 3 mL/min/1.73 m^2^ per year) in a cohort of 6790 participants (12% had diabetes but the type of diabetes is not reported) who all had preserved kidney function at baseline [30].

The exact pathophysiological link between weight and BMI variability and vascular damage remains unclear, but the association is likely bidirectional, indicative of a classic causality dilemma. While BMI oscillation may arise iatrogenically from aggressive weight control strategies, often referred to as ‘yo‐yo dieting’, their magnitude may also reflect underlying metabolic instability and as a causal factor, may contribute to oxidative stress, hypoxia, kidney hyperfiltration, inflammation, and dysfunctional adiposity [31, 32] which can all drive the progression of kidney disease.

Additionally, recurrent weight cycling is known to worsen lipid profiles and insulin resistance [31, 33]. Epigenetic changes induced by high BMI and increased weight variability may further exacerbate microvascular injury, underlining a complex interplay of factors [32]. An interesting concept in this context is the repeated ‘overshoot hypothesis’ which may explain the connections between BMI and body weight variability/cycling and adverse cardio‐renal‐metabolic outcomes. This hypothesis proposes that repetitive and sustained fluctuations in energy balance, which are likely to occur with BMI cycling, may also contribute to variability in other cardio‐renal determinants such as blood pressure, lipids, heart rate, and glycaemic measures such HbA1c. Indeed, the variabilities of different risk factors in people with T2DM or obesity can inter‐correlate, form clusters and synergistically impact the outcome [34, 35, 36, 37]. Further research that investigates the integrated impact and pattern of these indices and their variability on adverse clinical endpoints in pwT1DM is needed.

The association between visceral adiposity and microvascular diabetic complications may provide further mechanistic insight into our findings. In recent studies, visceral adiposity was associated with increased risk of albuminuria, peripheral neuropathy and cardiac autonomic neuropathy [38, 39, 40]. Ponirakis et al. observed that this association with peripheral neuropathy was mediated by elevated HbA1c levels, increased intima media thickness, systolic blood pressure, and triglycerides. Liu et al. showed in a population study of 2965 individuals, of which 10% had diabetes (both T1DM and T2DM) and 8% albuminuria, that in those with low visceral adiposity, diabetes was not significantly associated with albuminuria, while in participants with medium or high visceral adiposity, diabetes was an independent risk factor for albumunuria. Risi et al. observed that higher visceral adiposity was associated with increased risk of cardiac autonomic neuropathy and that this correlation was stronger in pwT1DM than in individuals with T2DM. These findings support the potential role of visceral adiposity in the pathogenesis of diabetic microvascular complications within the broader context of metabolic dysregulation. Indeed, there are emerging data that high BMI variability may lead to accumulation of visceral fat through loss‐regain cycles that may favour redistribution of adipose tissue as visceral fat [41, 42]. Further research is needed to investigate the underlying mechanisms of these associations and their relationship to the progression of diabetic kidney disease as well as other diabetes related microvascular complications.

With regard to the influence of ethnicity on DKD, the higher risk of kidney disease progression in pwT1DM of African‐Caribbean heritage, which we observed in our earlier studies, remained significant and independent of BMI variability indices [21, 26]. Socioeconomic deprivation, assessed using IMD decile, was not independently associated with DKD progression, and additional adjustment for IMD did not materially attenuate the associations between BMI variability and kidney disease progression outcome. However, we acknowledge that IMD as an index of soci‐socioeconomic deprivation has its limitations and other more nuanced and granular measures (e.g., education, income, occupation) would be required to fully exclude residual socioeconomic confounding [23].

Our results highlight the need for further research to explore potential mechanisms for our observations, which may be related to many factors including genetic predisposition, differences in metabolomic profiles, or variations in medication access and adherence [21, 26, 27].

The strengths of our study include its large, and more ethnically diverse cohort of pwT1DM than other studies in type 1 diabetes and the use of real‐world data over a long follow‐up period using standardised laboratory processes. We utilised multiple BMI indices of variability and our competing risk models demonstrated consistent effects of BMI variability on our clinically significant endpoint of advanced kidney disease. However, there are several limitations of our study; the retrospective nature of the study preclude causal inferences. Furthermore, the lack of data on specific confounders, such as insulin doses and adherence, renoprotective drug usage, dosage and adherence, is a further limitation. These could influence both BMI variability and DKD progression; therefore, their potential impact on our findings cannot be excluded. It is important to note however that BMI was associated with DKD progression independent of downstream markers associated with these confounders, such as HbA1c, systolic blood pressure and albuminuria. We note this limitation is often seen in similar large real‐world observational studies based on routinely collected electronic health record data, which are primarily captured for clinical care rather than research and often have incomplete ascertainment of medication exposure and limited ability to reliably measure dosing and adherence at scale [43, 44]. We observed that the significant, independent impact of BMI variability on kidney endpoints was not affected by measures of treatment response used in clinical care such as HbA1c, urine ACR and blood pressure; however, future studies with detailed treatment data including dosing, adherence and additional treatment measures are needed to further investigate whether our observed associations are influenced by these treatment/medication factors. Lack of information on eating patterns and eating disorders which impact BMI is another limitation of our study. Selection bias may also be present, as the cohort was recruited from specialised urban centres, potentially over‐representing high‐risk individuals. Larger studies with a wider population approach that address some of the above limitations are needed to further explore and confirm the generalisability of our results and that of the recent work in this area from the DCCT/EDIC cohort.

In conclusion, our study findings underscore the importance of considering BMI variability as a key risk factor for DKD in individuals with type 1 diabetes. These findings pave the way for further research and highlight the need for tailored interventions that account for both mean values and variability in managing diabetes‐related complications. Our results emphasise the necessity for clinicians to monitor not only the average levels of metabolic parameters but also their variability over time to better assess and manage the risk of adverse renal outcomes. Further research should focus on elucidating the biological mechanisms underlying the association between BMI variability and diabetic kidney disease. Our work also establishes the scientific rationale to further study whether interventions aimed at reducing BMI variability could translate to reduction in DKD progression.

## Author Contributions

J.K., M.O. and P.P. conceived the study. M.O., P.P., and J.K. wrote the manuscript. J.K. and M.O. developed the methodology and performed the literature investigation. S.A., J.K., P.P., M.A. and S.T. critically reviewed and edited the manuscript. M.O. and S.A. performed the statistical analysis. J.K. supervised the study. All authors have read and approved the final manuscript.

## Funding

This work was funded by a research grant from Guy's and St. Thomas Charity, London, U.K. (Grant JJ180101). S.A. was supported by the National Institute for Health Research Biomedical Research Centre based at Guy's and St. Thomas NHS Foundation Trust and King's College London. The views expressed are those of the authors and not necessarily those of the National Health Service, National Institutes of Health Research, or Department of Health.

## Conflicts of Interest

The authors declare no conflicts of interest.

## Supporting information


Supporting Information S1

Supporting Information S2

## Data Availability

The data that support the findings of this study are available from the corresponding author upon reasonable request.
