# Supplementary material for: Impact of Body Mass Index Variability on Kidney Disease Progression in a Large Type 1 Diabetes Cohort
Source: Diabetes Metab Res Rev. 2026 Mar 19;42(3):e70148. doi: 10.1002/dmrr.70148 (PMC13001802; doi:10.1002/dmrr.70148)
Supplement: Supplementary file 1 — Supporting Information S1 [file DMRR-42-e70148-s002.docx]

Cumulative incidence of the primary endpoint in BMI variability quartiles 1 (stable) and 4 (extreme fluctuators)


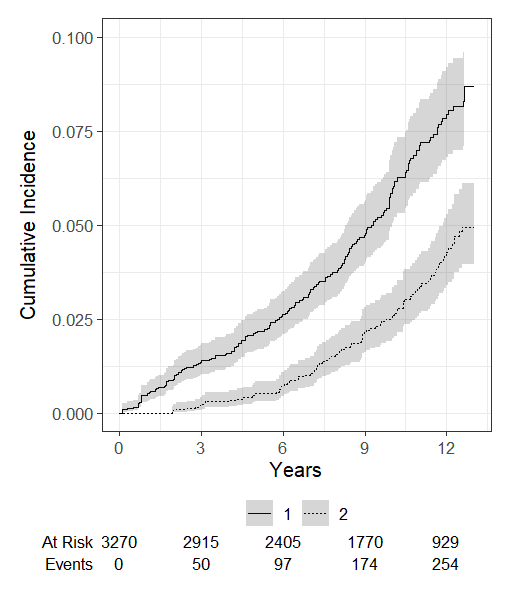


**Supplemental Figure SF1.** Cumulative incidence of the primary endpoint (≥50% decline in eGFR from baseline and a final eGFR <30 mL/min/1.73 m²) in the “stable” group in Quartile 1 (line 2) and “extreme fluctuators” in Quartile 4 (line 1)

eGFR=Estimated Glomerular Filtration Rate

Time-dependent AUC plots with BMI variability indices included in multivariable hazard models

**
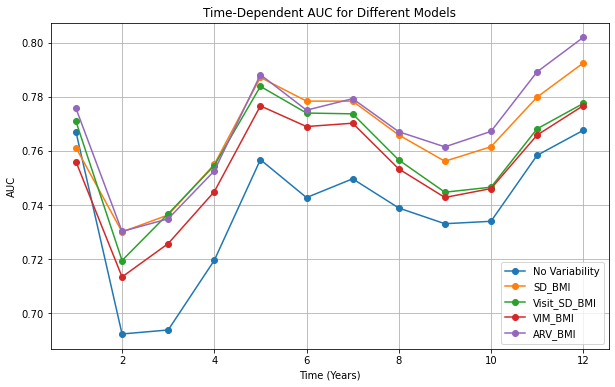
**

**Supplemental Figure SF2.**  Time-dependent AUC plots with BMI variability indices included in multivariable hazard models demonstrating consistently strong predictive performance over time. AUC= Area Under the Curve, ARV= Average Real Variability, SD= Standard Deviation, VIM= Variability Independent of the Mean
