# Supplementary material for: Impact of Body Mass Index Variability on Kidney Disease Progression in a Large Type 1 Diabetes Cohort
Source: Diabetes Metab Res Rev. 2026 Mar 19;42(3):e70148. doi: 10.1002/dmrr.70148 (PMC13001802; doi:10.1002/dmrr.70148)
Supplement: Supplementary file 2 — Supporting Information S2 [file DMRR-42-e70148-s001.docx]

Supplemental Table S1*: Comparison of BMI and BMI Variability Indices in people of African-Caribbean and Non-African Caribbean ethnicity*

|  | Ethnicity | |  |
| --- | --- | --- | --- |
| Variable | Non-African Caribbean Ethnicity | African-Caribbean Ethnicity | p-value |
| Baseline BMI (kg/m^2^) | 25.2 4.6 | 25.3 5.2 | 0.8 |
| Number of BMI measurements | 14 (13) | 16 (15) | 0.003 |
| log-SD | 0.76 (±0.32) | 0.84 (±0.32) | <0.001 |
| log-adj-SD | 1.6 (±0.57) | 1.75 (±0.6) | <0.001 |
| log-VIM | 1.64 (±0.68) | 1.8 (±0.68) | <0.001 |
| log-ARV | 0.54 (±0.22) | 0.57 (±0.2) | <0.001 |

*Abbreviations: BMI: Body Mass Index, log_SD: Log-transformed standard deviation of BMI, log_adj-SD: Log-transformed visit-adjusted standard deviation of BMI, log_VIM: Log-transformed variability independent of the mean for BMI, log_ARV: Log-transformed average real variability for BMI
†Parameters are presented as medians with interquartile ranges (IQR); comparison tests are performed using the Mann-Whitney U test.*

Supplemental Table S2*: Correlation between selected baseline covariates and BMI variability indices*

|  | **log_SD** | **log-adj-SD** | **log_ARV** | **log_VIM** |
| --- | --- | --- | --- | --- |
| **log_SD** | 1 | 0.903^**^ | 0.725^**^ | 0.872^**^ |
| **log_adj-SD** | 0.903^**^ | 1 | 0.516^**^ | 0.927^**^ |
| **log_ARV** | 0.725^**^ | 0.516^**^ | 1 | 0.551^**^ |
| **log_VIM** | 0.872^**^ | 0.927^**^ | 0.551^**^ | 1 |
| **Baseline eGFR** | 0.079^**^ | 0.076^**^ | -0.010 | 0.002 |
| **log_ACR** | 0.127^**^ | 0.066^**^ | 0.150^**^ | 0.059^**^ |
| **Baseline Age** | -0.141^**^ | -0.131^**^ | -0.071^**^ | -0.049^**^ |
| **Baseline SBP** | -0.045^**^ | -0.052^**^ | 0.004 | 0.060^**^ |
| **HBA1C** | 0.175^**^ | 0.166^**^ | 0.103^**^ | 0.111^**^ |
| **Baseline BMI** | 0.176^**^ | 0.114^**^ | 0.253^**^ | 0.440^**^ |

*** means significance at 0.001 level
Correlation coefficient levels: Weak <0.30, Moderate 0.3-0.7, strong > 0.7*

Supplemental Table S3: *Performance Evaluation of Multivariate Cox Proportional Hazard Models with BMI variability indices*

| Multivariate Cox Models Performance Metrics | | | | | |
| --- | --- | --- | --- | --- | --- |
|  | Model with no variability indices | Model with standard deviation | Model with visit adjusted standard deviation | Model with VIM | Model with ARV |
| Concordance Index | 0.742 | 0.775 | 0.771 | 0.768 | 0.774 |
| Likelihood ratio test | 158.8 | 200.8 | 203.5 | 198.2 | 192.8 |
| Akaike Information Criterion | 2591.8 | 2551.9 | 2549.2 | 2554.45 | 2559.9 |

*Abbreviations:VIM: Log-transformed variability independent of the mean for BMI, ARV: Log-transformed average real variability for BMI*

Supplementary Table S4. Sensitivity Analysis: multivariable Cox models integrating IMD

| Model | Variable | HR_CI | p_value |
| --- | --- | --- | --- |
| Baseline + IMD | Age (years) | 1.02 (1.01-1.04) | <0.001 |
|  | Baseline BMI (kg/m^2) | 1.01 (0.98-1.04) | 0.547 |
|  | Baseline eGFR (mL/min/1.73m^2) | 0.99 (0.98-0.99) | 0.001 |
|  | HbA1c (mmol/mol) | 1.02 (1.02-1.03) | <0.001 |
|  | log(ACR) | 1.34 (1.17-1.53) | <0.001 |
|  | Systolic BP (mmHg) | 1.01 (1.00-1.02) | 0.113 |
|  | African-Caribbean (vs non) | 1.74 (1.22-2.47) | 0.002 |
|  | **IMD decile (1=most deprived, 10=least)** | **1.03 (0.96-1.09)** | **0.436** |
| Visit-adj SD + IMD | log(visit-adjusted SD of BMI) | 2.37 (1.84-3.05) | <0.001 |
|  | Age (years) | 1.03 (1.01-1.04) | <0.001 |
|  | Baseline eGFR (mL/min/1.73m^2) | 0.98 (0.97-0.99) | <0.001 |
|  | HbA1c (mmol/mol) | 1.02 (1.02-1.03) | <0.001 |
|  | log(ACR) | 1.31 (1.15-1.50) | <0.001 |
|  | Systolic BP (mmHg) | 1.01 (1.00-1.02) | 0.064 |
|  | African-Caribbean (vs non) | 1.78 (1.26-2.53) | 0.001 |
|  | **IMD decile (1=most deprived, 10=least)** | **1.01 (0.95-1.08)** | **0.694** |
| ARV + IMD | log(ARV of BMI) | 5.08 (3.08-8.37) | <0.001 |
|  | Age (years) | 1.02 (1.01-1.04) | <0.001 |
|  | Baseline eGFR (mL/min/1.73m^2) | 0.99 (0.98-0.99) | 0.001 |
|  | HbA1c (mmol/mol) | 1.02 (1.02-1.03) | <0.001 |
|  | log(ACR) | 1.27 (1.11-1.46) | <0.001 |
|  | Systolic BP (mmHg) | 1.01 (1.00-1.02) | 0.068 |
|  | African-Caribbean (vs non) | 1.86 (1.31-2.65) | <0.001 |
|  | **IMD decile (1=most deprived, 10=least)** | **1.02 (0.96-1.09)** | **0.454** |

*Abbreviations: BMI: Body Mass Index, log_SD: Log-transformed standard deviation of BMI, log_adj-SD: Log-transformed visit-adjusted standard deviation of BMI, log_VIM: Log-transformed variability independent of the mean for BMI, log_ARV: Log-transformed average real variability for BMI, IMD: Index of Multiple Deprivation, BP: Blood Pressure, ACR: Albumin Creatinine Ration, eGFR: Estimated Glomerular Filtration Rate*
